# Detection of SARS-CoV-2 in the Indoor and Outdoor Areas of Urban Public Transport Systems of Three Major Cities of Portugal in 2021

**DOI:** 10.3390/ijerph19105955

**Published:** 2022-05-13

**Authors:** Priscilla Gomes da Silva, José Gonçalves, Maria São José Nascimento, Sofia I. V. Sousa, João R. Mesquita

**Affiliations:** 1 ICBAS—Instituto de Ciências Biomédicas Abel Salazar, School of Medicine and Biomedical Sciences, University of Porto, 4050-313 Porto, Portugal; 2 Epidemiology Research Unit (EPIUnit), Instituto de Saúde Pública, University of Porto, 4050-600 Porto, Portugal; 3 Laboratório para a Investigação Integrativa e Translacional em Saúde Populacional (ITR), 4050-313 Porto, Portugal; 4 LEPABE—Laboratório de Engenharia de Processos, Ambiente, Biotecnologia e Energia, Laboratory for Process Engineering, Environment, Biotechnology and Energy, Faculty of Engineering, University of Porto, 4200-465 Porto, Portugal; sisousa@fe.up.pt; 5 Department of Chemical Engineering and Environmental Technology, Institute of Sustainable Processes, University of Valladolid, 47011 Valladolid, Spain; zemcg5@gmail.com; 6 Faculty of Pharmacy, University of Porto, 4050-313 Porto, Portugal; saojose@ff.up.pt; 7 ALiCE—Associate Laboratory in Chemical Engineering, Faculty of Engineering, University of Porto, Rua Dr. Roberto Frias, 4200-465 Porto, Portugal

**Keywords:** SARS-CoV-2, air sampling, airborne transmission, environmental contamination, indoor air, outdoor air

## Abstract

Airborne transmission is mainly associated with poorly ventilated and crowded indoor environments where people stay for long periods of time. As such, public transport is often perceived as having a high risk for the transmission of SARS-CoV-2. Considering that data on the detection of SARS-CoV-2 in public transport systems are scarce, we performed air sampling for SARS-CoV-2 in indoor and outdoor spaces of public transport systems in Portugal. Air (*n* = 31) and surface (*n* = 70) samples were collected using a Coriolis^®^ Compact microbial air sampler and sterile flocked plastic swabs, respectively. Samples were extracted and analyzed through RT-qPCR. Only two air samples from an outdoor and a partially open space were found to be positive for SARS-CoV-2 RNA. No positive surface samples were detected. These results indicate that the viral concentration in ambient air in public transport systems is linked to the number of people present in that environment and whether they are wearing properly fitting masks. Considering the current lifting of COVID-19 restrictions around the world, it is essential that people continue to wear masks in both indoor and outdoor environments, especially in crowded spaces. More studies on this topic are needed to fully elucidate the real risk of infection in outdoor spaces.

## 1. Introduction

The main modes of transmission for respiratory viruses are reported in the literature as direct, via physical contact between an infected individual and a susceptible individual; indirect, via contact with contaminated surfaces or objects (fomites); or through the air via contaminated aerosols that might be suspended and therefore inhaled by susceptible people [1]. Regarding SARS-CoV-2, much attention was given to the fomite transmission route and the cleaning of surfaces in the first year of the pandemic. However, it is widely accepted that COVID-19 rarely spreads through surfaces [2], with the focus turning to the airborne transmission route as the key way SARS-CoV-2 has spread worldwide [3].

Now, two years since the pandemic started, airborne transmission is widely recognized as a major mode of transmission for SARS-CoV-2 [4,5], and is mainly associated with poorly ventilated indoor environments [6], especially crowded indoor environments where people might spend long periods of time [7]. This is particularly relevant in public transport, with people who need to use these systems to commute to work showing concern about their safety during the pandemic [8]. It is noteworthy that it has been reported that the chances of those using public transport acquiring an acute respiratory infection increased up to six times during influenza outbreaks [9]. As such, public transport is often perceived as posing a higher risk for the transmission of respiratory viruses. These environments are usually crowded and it is more difficult to maintain social distancing in them [1,10]. Consequently, a significant change in people’s mobility patterns and usage of public transport systems was seen during the pandemic, as people started to avoid using public transport systems for commuting to reduce their risk of infection with COVID-19 [11,12,13].

Despite this, only a few studies have been published on SARS-CoV-2 transmission in public transport systems. An study conducted by Luo et al., 2020 [14], reported a SARS-CoV-2 outbreak event linked to bus journeys, where 234 individuals were epidemiologically linked to the bus journeys of a person infected with COVID-19, of which 12 tested positive for COVID-19. Another study carried out by Harris et al, 2020 [15], reported that the New York City’s subway system played a significant role in SARS-CoV-2 transmission during the first wave of the disease in March 2020.

Moreover, regarding the environmental contamination and presence in the air of SARS-CoV-2 in public transport systems, only three studies where viral RNA presence was detected have been published so far, to the best of our knowledge. The first one, by Di Carlo et al., 2020 [16], assessed the presence of SARS-CoV-2 RNA in the air and on surfaces frequently touched by passengers inside a city bus during its normal operation. However, after two weeks of measurements, the virus was not detected in any sample. In the study by Troko et al., 2021 [8], the presence of SARS-CoV-2 RNA in the air and on surfaces on public buses and subway trains was assessed, with viral presence being detected in both surface and air samples. The study conducted by Comunian et al., 2021 [17], collected aerosol samples from both indoor (hospital facilities) and outdoor public spaces, including a bustling bus station. Although SARS-CoV-2 RNA was detected in the indoor areas studied, no viral presence was found in the bus station. Nevertheless, due to experimental limitations, only the presence of viral RNA was assessed in all these studies and viral viability could not be studied; thus, no assumptions could be made regarding whether the virus was infectious or not [18].

Considering that data on the detection of SARS-CoV-2 in public transport systems are still scarce, this study aimed to perform air sampling and tests for SARS-CoV-2 in the indoor and outdoor spaces of public transport systems in three major cities of Portugal during the period following the 3rd wave of lockdown release. During this time, the effective reproduction number R(t) for SARS-CoV-2 was 1.03 in the northern region and 1.00 in the central region of Portugal, respectively [19]. Moreover, considering that virus-laden aerosols that might have been suspended in the air could have already settled onto surfaces prior to our sampling, we have also performed surface sampling inside public transport vehicles.

## 2. Materials and Methods

### 2.1. Sampling Sites

Air and surface sampling was performed between 5 April 2021 and 30 April 2021 in the indoor and outdoor areas of the urban public transport systems of three major cities of Portugal, one from the northern region (City A) and two from the central region (City B and C). Air samples (*n* = 31) were collected in 4 different train stations and 15 bus stations during the rush hour in the morning, between 7 and 9 am. Surface samples (*n* = 70) were collected inside 14 different trains during their journeys to and from the three cities between 6 and 12 am. Details of the sampling sites can be found in Table 1.

### 2.2. Collection of Air and Surface Samples

Air samples were collected using the Coriolis^®^ Compact (Bertin Instruments, Montigny-le-Bretonneux, France) cyclonic microbial air sampler with an airflow rate of 50 L/min (total of 1.5 m^3^) for 30 min. The sampler was placed at an approximately 1 m height in every sampling site. Air samples were collected on a dry medium, with 4 mL of sterile phosphate-buffered saline (PBS) added to the sterile collection cones after each sampling. After every sampling, the cleaning and decontamination of the sampler were performed according to the manufacturer’s instructions. Briefly, a wipe dampened with a surfactant–water solution was used to clear the external parts of the Coriolis compact. After that, the sampler was wiped down with a soft cloth to remove any excess.

Surface samples were collected on 10 cm × 10 cm surface areas (100 cm^2^) using sterile flocked plastic swabs previously wetted on PBS and immediately placed in vials containing 4 mL of PBS. All samples were stored at 4 °C in sterile 1.5 mL RNAse-free Eppendorfs until transportation to the laboratory facilities and processed within 24 h in order to ensure the minimum loss of viral RNA.

It should be noted that the person performing the sampling was wearing KN95 masks and gloves at all times, with their mask being changed every 4 h, their gloves being changed after handling each sample, and their hands being washed between handling each sample.

### 2.3. RNA Extraction and RT-qPCR

RNA extractions were performed using the GRS Viral DNA/RNA Purification Kit (GRISP, Porto, Portugal) according to the manufacturer’s instructions. RNA extraction was performed using 200 μL of sample suspension, as previously described [20]. A one-step RT-qPCR reaction targeting two viral gene targets (N1 and N2) was used (Xpert qDetect COVID-19, GRISP, Porto, Portugal). Selected targets, corresponding primer sequences, and probes were chosen based on a previously described protocol [21]. The CFX Real-Time PCR (qPCR) Detection System (Bio-Rad, Hercules, CA, USA) with the CFX Maestro 1.0 Software version 4.0.2325.0418 (Bio-Rad, Hercules, CA, USA) was used to control the runs and remotely analyze the data. Each RT-qPCR run included ssDNA targets for both the N1 and N2 regions (positive controls) and a no-template control. The PBS used for the collections was also tested through RT-qPCR in order to show that no virus was present in it and therefore exclude the possibility of false positives through the contamination of the suspension media. Reactions were set up and run with the initial conditions of 15 min at 45 °C and 2 min at 95 °C, then 45 cycles of 95 °C for 15 s and 55 °C for 30 s. A standard curve was generated using ssDNA targets for both the N1 and N2 regions in a 10-fold serial dilution mixture starting at 200,000 copies/µL in order to quantify the number of viral gene copies present in each sample from the measured Ct values, with limit of detections (LODs) of 1.3 copies/µL for N1 and 3.2 copies/µL for N2. Air sample results are expressed in copies/m^3^ and surface sample results in copies/cm^2^.

## 3. Results

During the sampling period of this study, Portugal was between two waves of COVID-19 outbreaks and the rate of transmission of SARS-CoV-2 was considered low, with a national incidence of 64.3 cases of COVID-19 per 100,000 inhabitants [22]. The effective reproduction number R(t) for SARS-CoV-2 was 1.03 and 1.00 for Portugal’s northern and central regions, respectively [19].

Of the 31 air samples collected in the public transport systems, only two (both from the train station of City A, one collected in front of the station (open outdoor area) and the other in the entrance hall (partially open)) were positive for SARS-CoV-2 RNA, which corresponds to a positivity of 9.5% (2 out of 21) of the outdoor air samples, compared to 0% (0 out of 10) of the indoor air samples. Both positive air samples were taken on the same day, with an interval of ~10 min between each other. The sample from the front of the train station was amplified for target genes N1 (8500 gene copies/m^3^) and N2 (6025 gene copies/m^3^), while the sample from the entrance hall of the train station was amplified only for N2 (6312 gene copies/m^3^). Positivity for only one region has often been reported in other studies, which is likely due to primer mismatching [23].

Of the 70 surface samples collected in different trains going to and coming from the three cities, none were positive for SARS-CoV-2 RNA.

## 4. Discussion

Studies on SARS-CoV-2 in outdoor public locations are scarce and the results are contradictory, with some reporting high rates of SARS-CoV-2 RNA positivity in these environments [8] and others reporting no viral presence at all [16,17,24,25]. In the present study, out of 31 air samples collected in public transport systems, only 2 were found to be positive for SARS-CoV-2 RNA, with all surface samples being negative for the presence of viral RNA. Regarding surface samples, this result is not unexpected considering the infection prevention measures enforced in Portugal at the time, in which train surfaces were disinfected before passengers were allowed to go in, added to the fact that all passengers were wearing surgical or KN95 masks at all times when inside the trains, therefore decreasing the probability of viral shedding to the environment inside trains. The two positive samples were taken from City A at the same train station, with the first sample being taken in an outdoor open space. This train station is located at the center of the City A and is composed of an entrance hall connected to the train platforms in the back, which are in the open air. Moreover, the main entrance doors remain open, therefore allowing for constant natural ventilation within the station. These two samples were taken in the morning in the rush hour—i.e., between 7 and 9 am—when the train station is crowded and is more full of people due to work commutes. In this case, the large number of people passing by during the sampling period can explain the detection of SARS-CoV-2 in the air. The presence of SARS-CoV-2 in public spaces is reported to be directly related to the number of infected people in that environment—i.e., the more people are present, the more likely it is that high viral concentrations of the virus might be present [26,27]. It is of note that the 14-day cumulative incidence rate per 100,000 inhabitants in the northern region of the country, where City A is located, was 64.7 when the positive sample was collected.

The first issue to be considered when interpreting the discrepant results of our study and previous studies on this subject is the lack of a standard methodology for sampling air when it comes to SARS-CoV-2. Additionally, different research groups use different parameters in their sampling, such as airflow rate, sampling duration, sample storage, and air sampler type, which should be taken into consideration. The air sampler itself is a very important factor, as air samplers with different collection methodologies (cyclone, impinger, impactor, filter, water condensation) might have different efficiency recovery rates for sampling SARS-CoV-2. Second, it should be considered that the sampling protocols used in these studies are implemented in different countries and/or cities and at different months throughout 2020 and 2021, which means that the meteorological conditions and epidemiological situation in each studied location will vary greatly. Furthermore, no control samples were used during our experiment. as the air sampling took place in indoor and outdoor public spaces where people were present at all times; therefore, it was not possible to perform air sampling in a controlled environment with clean air.

The global positivity in this study was low. Notwithstanding, the spaces where viral RNA was detected in air were either partially open (entrance hall of the train station) or completely open to the outdoor air (front of the train station), where more people not wearing masks were present, in contrast to the air of closed spaces where everyone was wearing a mask at all times and no positive surface sample could be detected. The presence of viral RNA in outdoor air, where ventilation is optimal, suggests that the presence of SARS-CoV-2 in the air is not linked to proper ventilation but rather to the number of people present in that environment and if they are wearing properly fitting masks or not, especially considering that no viral RNA could be detected in the public indoor spaces of the urban public transport systems, where, according to Decree-Law No. 78-A/2021 in force in Portugal at the time, the use of proper masks (of surgical grade or higher) was mandatory, with penalties that varied between EUR 100 and 500 for those who failed to follow the law, reinforcing the notion that the proper use of masks greatly decreases the amount of virus emitted to the environment.

It is important to highlight the value of maintaining preventive measures such as the regular disinfection of public transport system areas, proper ventilation, good hand hygiene, and wearing well-fitting masks at all times. However, even though the risk of infection in these environments is potentially low, the results presented here show that the risk of airborne transmission of COVID-19 in public transports exists in certain circumstances—namely, when infection prevention measures such as proper ventilation, wearing appropriate masks, and keeping at least 1 m of space between people are not strictly followed. Additionally, caution is needed when interpreting the results of this study because positive samples do not necessarily correlate to infectious SARS-CoV-2 particles. Moreover, even if viable particles were present, the viral loads found in the air could have been below the minimum infective dose of SARS-CoV-2 needed to cause COVID-19.

## 5. Conclusions

In this study, we report the detection of SARS-CoV-2 RNA in the outdoor environments of the urban public transport systems of Portugal. These outdoor environments were crowded and the majority of people were not wearing protective masks. Considering the current lifting of COVID-19 restrictions all around the world, it is essential that people continue to wear masks in both indoor and outdoor environments, especially in crowded spaces, as wearing a mask decreases the risk of transmission through air. More studies including data about viral viability, duration of exposure, frequency of exposure, number of people wearing masks, and distribution of virus-laden particles of different sizes are needed to fully elucidate the real risk of infection in outdoor spaces.

## Figures and Tables

**Table 1:** Details of air and surface sampling sites in the public transport systems of three major cities in Portugal, 2021.

	Cities (Region)	Public Transport Systems (Total Number)	Sample Locations (Number of Samples)	Sampling Location Type	Total Number of Samples
Air sampling	City A (North)	Train station (*n* = 2)	Entrance hall (*n* = 1)	Indoor	13
			Waiting room (*n* = 1)	Indoor	
			Middle platform (*n* = 1)	Outdoor	
			Front of the station (*n* = 1)	Outdoor	
		Bus station ^a^ (*n* = 5)	Bus stop (*n* = 1)	Outdoor	
	City B (Center)	Train station (*n* = 1)	Entrance hall (*n* = 1)	Indoor	9
			Waiting room (*n* = 1)	Indoor	
			Middle platform (*n* = 1)	Outdoor	
			Front of the station (*n* = 1)	Outdoor	
		Bus station ^a^ (*n* = 5)	One sample in each for a total of 5 samples	Outdoor	
	City C (Center)	Train station (*n* = 1)	Entrance hall (*n* = 1)	Indoor	9
			Waiting room (*n* = 1)	Indoor	
			Middle platform (*n* = 1)	Outdoor	
			Front of the station (*n* = 1)	Outdoor	
		Bus station ^a^ (*n* = 5)	One sample in each station (*n* = 1)	Outdoor	
Surface sampling	City A to City B City B to City A City B to City C City C to City B	Trains (*n* = 14)	Window (*n* = 1) Arm rest (*n* = 1) Grab handles ^b^ (*n* = 2) Door button (*n* = 1)	Indoor	70

^a^ Bus stations were single-bus stations in central areas of the city. ^b^ Handles are located on the top of seats so that people travelling standing up can use them to hold on safely while on the train.

## Data Availability

Not applicable.
